# Predictors of Step Length from Surface Electromyography and Body Impedance Analysis Parameters

**DOI:** 10.3390/s22155686

**Published:** 2022-07-29

**Authors:** Jin-Woo Park, Seol-Hee Baek, Joo Hye Sung, Byung-Jo Kim

**Affiliations:** 1 Department of Neurology, Korea University Anam Hospital, Korea University Medicine, Seoul 02841, Korea; parkzinu@korea.ac.kr (J.-W.P.); virgo0906@korea.ac.kr (S.-H.B.); centertruth@korea.ac.kr (J.H.S.); 2 Division of Clinical Pharmacology, Vanderbilt University Medical Center, Nashville, TN 37232, USA; 3 BK21 FOUR Program in Learning Health Systems, Korea University, Seoul 02841, Korea

**Keywords:** step length, surface electromyography, body impedance analysis

## Abstract

Step length is a critical hallmark of health status. However, few studies have investigated the modifiable factors that may affect step length. An exploratory, cross-sectional study was performed to evaluate the surface electromyography (sEMG) and body impedance analysis (BIA) parameters, combined with individual demographic data, to predict the individual step length using the GAITRite^®^ system. Healthy participants aged 40–80 years were prospectively recruited, and three models were built to predict individual step length. The first model was the best-fit model (R^2^ = 0.244, *p* < 0.001); the root mean square (RMS) values at maximal knee flexion and height were included as significant variables. The second model used all candidate variables, except sEMG variables, and revealed that age, height, and body fat mass (BFM) were significant variables for predicting the average step length (R^2^ = 0.198, *p* < 0.001). The third model, which was used to predict step length without sEMG and BIA, showed that only age and height remained significant (R^2^ = 0.158, *p* < 0.001). This study revealed that the RMS value at maximal strength knee flexion, height, age, and BFM are important predictors for individual step length, and possibly suggesting that strengthening knee flexor function and reducing BFM may help improve step length.

## 1. Introduction

Walking performance is an important hallmark of evaluating individual health status [1,2,3]. The slow progressive decline in walking performance is associated not only with neurodegenerative disorders (e.g., Alzheimer’s disease and Parkinson’s disease) [4,5,6,7], but also with aging [8]. The commonly used parameters of walking performance include gait speed, cadence, cycle time, and stride length [9,10]. Among these, step length, defined as the distance between the initial contact point of one foot and the initial contact point of the opposite foot, has been widely used for general gait analysis, sports training, and medical rehabilitation [2,11,12]. In particular, shortened step length in the elderly is an indicator of the risk of functional loss and falls [13]. However, the effects of modifiable factors in terms of musculoskeletal function and the components of body weight on step length have not yet been explored.

Among the leg muscles involved in gait performance, the thigh muscles have been the primary focus. Site-specific sarcopenia was more frequently found in the thigh muscles than in the calf muscles as part of the normal aging process [14,15], and age-related loss of thigh muscle was related to difficult task performance (e.g., zig-zag walking) [14].

Various methods have been used previously to evaluate muscle function and quality. These include surface electromyography (sEMG) and body impedance analysis (BIA), which have been commonly used in clinics and in various fields of research [16,17]. sEMG is a non-invasive approach that collects data reflecting muscle activity in dynamic conditions. BIA is another method for measuring body composition; it has many merits (e.g., lower cost, no radioactivity) compared with dual-energy X-ray absorptiometry (DXA), which has been considered a gold standard for body composition assessment.

Therefore, this study was performed to evaluate the value of various parameters for predicting individual step length using sEMG (focused on the thigh muscles) and BIA combined with individual demographic data.

## 2. Materials and Methods

### 2.1. Study Design and Participants

An exploratory cross-sectional study was performed to examine the relationship among step length, sEMG parameters, and body composition. Healthy individuals aged 40–80 years were prospectively recruited. Participants were eligible when their medical research council scale grade at the time of participation in the study was five in all extremities. Participants with a history of any health problem that may cause muscle weakness and/or gait difficulty within 3 months and any sequelae related to muscle weakness or gait-related problems were excluded. This study was approved by our Institutional Review Board (IRB number 2020AN0361), and informed consent was obtained from all participants.

### 2.2. Step Length Measurement

Step length was measured using the GAITRite^®^ system (CIR Systems, Inc., Franklin, NJ, USA), a commercially available 4.27 m long electronic walkway comprising multiple sensor pads inserted in a grid formation between layers [18,19,20]. The sensors were activated by mechanical pressure, and the signals (sampling rate of 80 Hz) were transferred to the computer and stored. The participants were instructed to walk at their most comfortable and regular gait speed on the walkway. The average step length (cm) was measured as the line of progression from the heel center of the current footprint to the heel center of the previous footprint of the opposite foot. Next, the average value of every step length measured from each step (right and left sides) was used for statistical analysis.

### 2.3. sEMG Measurement

sEMG signal data from the participants’ lower extremities were obtained using the Nicolet EDX system (Natus Neurology Inc., Middleton, WI, USA) and Synergy (Synergy Healthcare Solutions, Maryville, TN, USA). The sEMG signals were amplified and band-pass filtered at 100–500 Hz at a sampling rate of 48 kHz, and raw sEMG signals were full-wave rectified. Three recording electrodes (diameter: 20 mm, Natus Neurology Inc., Middleton, WI, USA) were linearly (perpendicular to the axis of the lower extremity) attached to each muscle belly of the rectus femoris (representing the extensor muscle) and biceps femoris (representing the flexor muscles of the knee joint). Six electrodes were attached to the thigh to minimize factors affecting signal acquisition (e.g., diameter, position, and depth of the muscle fiber) with a 10 mm interelectrode distance. A reference electrode was placed on the lateral prominence of the patella during knee flexion and extension, and a ground electrode was placed close to the recording electrodes. Next, the participant was asked to lie down in a prone position and to maximally flex and extend his/her knee joint against the examiner’s resistance to monitor continuous maximal voluntary contraction using the manual muscle tester 01165 (Lafayette Instrument Company, Lafayette, LA, USA). After the test trials, participants were required to flex or extend their legs at their maximal effort for 3 s against the investigator’s resistance. The electrical activity of the muscle from each electrode was selected (from the biceps femoris at flexion and rectus femoris at extension), and was calculated by the root mean square (RMS) value over the period of resistance (Figure S1). The average RMS value from the three electrodes on the rectus femoris (during extension) and biceps femoris (during flexion) was used for statistical analyses.

### 2.4. BIA

Body fat mass (BFM, kg) and appendicular skeletal muscle mass (ASM, kg) were measured using a multifrequency 80-electrode body impedance analyzer (Inbody770^®^ system, InBody Corp, Seoul, Korea). The Inbody770^®^ system has been commonly used in previous research for measuring individual body components [21,22,23] and the validation of its use has been described previously [24,25,26,27,28]. Thirty impedance measurements were obtained at six different frequencies (1, 5, 50, 250, 500, and 1000 kHz) for five body segments (both arms and legs and trunk). The measurements were performed at least 3 h after a meal, and participants were instructed not to exercise intensively before the examination to minimize other factors that would affect the results. The ASM and BFM values were calculated using the manufacturer’s algorithm [21]. The calculated ASM results were divided by the squared height (m^2^) to adjust for the effect of height on ASM, and these values were used for statistical analysis.

### 2.5. Statistical Analysis

Analysis of variance was used to compare the demographics and results of BIA and sEMG measurements between each age group. The *t*-test was used to compare step length differences by sex. Simple linear regression analysis was used to assess the associations between the variables and identify more significant variables related to step length, and scatter plots were obtained. Multivariable regression with stepwise selection was used to obtain regression models, which were used to estimate the average step length. Statistically significant variables from the simple linear regression analysis and *t*-test were included in the multivariable regression analysis. The Shapiro–Wilk and Durbin–Watson tests were performed to check the assumptions of normality and independence (Ps > 0.05). The collinearity among multiple predictors was evaluated by variance inflation factors (VIF) and tolerance levels. The VIFs were below 1.06 and tolerance levels were above 0.942 for all variables. R squared (R^2^) and adjusted R^2^ were introduced to compare the results of the regression analyses between the variables and models. All or some of the variables were used for the three models based on the availability of BIA and sEMG. The first model included all candidate variables for the analysis. The second model used all candidate variables, except sEMG variables, with the assumption that the sEMG measurements were not available. The third model used all candidate variables except for sEMG and BIA variables. Statistical significance was set to *p* < 0.05. All statistical analyses were performed using SAS software (version 9.4, SAS Institute, Inc., Cary, NC, USA). The step length estimated by the models and the actual average step length measured by the GAITRite^®^ system from each individual were compared using linear regression.

## 3. Results

The baseline characteristics of the participants and a summary of the results are presented in Table 1.

**Figure 1:**
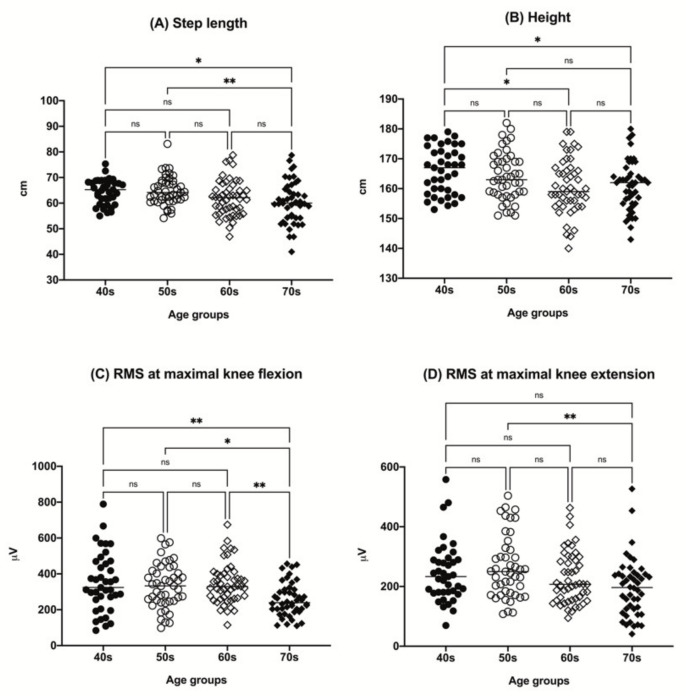
The distributions of participant step length (**A**), height (**B**), RMS value at maximal knee flexion (**C**), and RMS value at maximal knee extension (**D**) by age group (40s, age 40–49 years; 50s, age 50–59 years; 60s, age 60–69 years; 70s, age 70–79 years). * *p* < 0.05, ** *p* < 0.01. ns, not significant; RMS, root mean square.

A total of 179 participants (82 men (45.8%) and 97 women (54.2%)) completed the study. The participants’ demographics and differences in the variables based on the age groups and sex are shown in Table 2 and Figure 1.

There was a tendency for a decrease in height with increasing age among the groups. The step length tended to decrease in the older age group. A tendency for a decrease in RMS value with aging in both maximal knee flexion and extension was observed (Figure 1). Other parameters, including sex, body mass index (BMI), ASM/(height, m)^2^, and BFM, did not show any significant differences among the age groups.

Men were taller in all age groups. Although there was no significant difference in the average step length based on sex in the 40s age group, men showed a longer average step length in the other age groups. Whereas the ASM/(height, m)^2^ was higher in men across all age groups, there was no difference in the BFM, except in the 70s age group. No sex differences were found in the average RMS value at maximal knee extension across all age groups. However, the average RMS value at maximal knee flexion was higher among men in the 60s and 70s age groups (Table 2). Sex differences in the step length, height, average RMS value at knee flexion, and BFM across all ages are shown in Table 3.

Results of the simple linear regression analysis between the average step length and the variables (age, height, BMI, ASM/(height, m)^2^, and RMS values) are shown in Figure S2.

Among the variables, height (*p* < 0.0001, R^2^ = 0.1282) and RMS value at maximal knee flexion (*p* = 0.0001, R^2^ = 0.1632) showed the highest positive correlations with step length. The average step length had a weak negative correlation with age (*p* = 0.007, R^2^ = 0.0672) and BFM (*p* = 0.031, R^2^ = 0.0480), whereas the ASM/(height, m)^2^ (*p* = 0.0024, R^2^ = 0.0507) and RMS value at maximal knee extension (*p* < 0.0001, R^2^ = 0.0854) had relatively low positive correlations. BMI was not significantly correlated with step length. There was a significant difference in the average step length by sex (men: 64.83 ± 6.91 cm, women: 61.07 ± 6.25 cm, *p* = 0.012).

Based on the results of the simple linear regression analysis, we assumed that height and RMS value at maximal strength knee flexion were the major variables for estimating the individual average step length. The developed step length estimation models are presented in Table 4.

Age, sex, height, BFM, ASM/(height, m)^2^, and RMS value at maximal knee flexion and extension were selected as candidate variables for the multivariable regression analysis. The first model was the best-fit model using all statistically significant variables (R^2^ = 0.244, adjusted R^2^ = 0.235, *p* < 0.001). The RMS value at maximal knee flexion and height were included in this model (*p* < 0.001). The second model revealed that age, height, and BFM were significant variables for predicting the average step length (R^2^ = 0.198, adjusted R^2^ = 0.185, *p* < 0.001). Finally, the third model that was intended to predict the step length in the absence of sEMG and BIA showed that only age and height remained significant (R^2^ = 0.158, adjusted R^2^ = 0.148, *p* < 0.001).

## 4. Discussion

In this study, we demonstrated that height, age, RMS value at maximal strength knee flexion, and BFM are statistically significant factors that affect individual step length. The discovery of the RMS value at maximal strength knee flexion and BFM as step length determinants is meaningful because they are considered modifiable factors that may increase step length. In other words, better knee flexor function and a lower BFM may help increase individual step length.

Height, age, fat mass, and lower extremity muscle strength have been suggested to affect individual gait performance and velocity [29,30,31,32]. However, the use of sEMG parameters to predict individual step length has not been previously attempted. Our results demonstrated that the sEMG RMS value of the biceps femoris at knee flexion with maximal strength and height had the greatest influence on step length (R^2^ = 0.244, adjusted R^2^ = 0.235, *p* < 0.001).

As previous research has demonstrated the correlations between step length and step frequency with lower limb muscle function [33], we intended to determine the correlations between step length and the sEMG parameters during maximal strength knee joint flexion and extension (Figure S2). Among the variables, step length, measured at individuals’ regular walking velocity, showed the highest correlation with the RMS value obtained from the knee flexor during maximal flexion effort (R^2^ = 0.1632, *p* < 0.0001). These results were plausible and consistent with previous reports, suggesting that knee flexion strength is an important predictor of gait performance [34]. Consistent with this observation, Mañago et al., discovered in their multivariable analysis that knee flexion strength remained a significant variable for predicting gait speed, whereas knee extension did not [35]. They explained that this phenomenon was possible because knee flexors are most active in normal gait, and they may help prevent hyperextension in the standing position [35]. We also observed that gait speed was highly correlated (positively) with step length (R^2^ = 0.686, *p* < 0.001, Figure S3). Gait speed is known to be affected by lower extremity power [36]. Therefore, it is reasonable to assume that knee flexion strength is an important predictor of step length.

sEMG is used to detect electrical signals generated by activated muscle fibers [37]. The amplitude, time, and frequency domains of the sEMG signals are affected by the timing and intensity of the muscle contraction [38]. The RMS value, calculated from the average power of these signals, is used as a validated parameter from sEMG data in many types of research [39,40,41]. Although the RMS value at maximal knee flexion strength tended to positively correlate with measured strength, it did not show a very high correlation in our study (R^2^ = 0.1549, *p* < 0.001, Figure S4). Whether there is a linear relationship between muscle strength and sEMG signals remains unclear [42]. The sEMG signals may reflect the electrical activity of the flexor or extensor muscles during voluntary contraction rather than muscle strength, which involves more complex steps, including the electrical–contraction coupling of muscles. Among the sEMG parameters, we used the RMS values because they are widely used and reflect the intensity and duration of the sEMG signals during muscle contraction [43]. Therefore, our results imply that sEMG, specifically the RMS value at maximal strength knee flexion, can be a reliable functional marker for individual gait performance, including step length.

BFM is also a reasonable predictor of step length. Studies have demonstrated that BFM is negatively correlated with physical performance and balance, and limited lower extremity performance and endurance in the elderly, indicating the detrimental effect of high BFM on walking [44,45]. This is consistent with our results, which showed that a shortened step length was associated with higher BFM.

Although a previous study suggested a sex difference in step length [46], and our data also showed some differences, no significant difference was found in our models. The step length was slightly longer in men than in women (men: 64.83 cm vs. women: 61.09 cm, *p* < 0.001) in our data (Table 3). We assumed that the effect of sex was masked by height (men: 169.30 cm vs. women: 157.22 cm, *p* < 0.001). The RMS values at knee flexion (men: 252.51 vs. women: 216.57, *p* < 0.001) and BFM (men: 17.79 vs. women: 19.85, *p* = 0.036) variables were included in the final models.

Our comparison of the measured parameters between the age groups revealed that RMS at maximal knee flexion abruptly decreased in the 70s age group, which may also have effected the decreased step length in participants the age of 70. Moreover, the inter-individual step length difference was the highest in the older age group (up to 13.7%). Considering that individual step length may reflect the flexor muscle functional status, it is presumable that strengthening flexor muscle function would be beneficial for improving gait function, especially in the older population.

The beneficial effects of increasing step length on the health parameters, such as blood pressure, exercise capacity, and quality of life, have been investigated in many previous studies [47]. Our results suggest the possibility that strengthening the thigh muscle, the flexor muscles in particular, and reducing body fat mass may be helpful for increasing step length.

Some limitations should be acknowledged. First, the R^2^ values found in our models were relatively small, and the models were not aimed to measure ‘accurate step length’ for each individual. However, the results are still sufficient to suggest that knee flexor function and BFM may affect individual step length. We believe that further elaborated research, such as joint angle measurements during the gait may advance our models [48]. Second, sEMG data from the lower part of the leg (e.g., tibialis anterior and gastrocnemius) were not included in this study. Third, this study included only the Korean population. Considering the ethnic differences in body proportion (e.g., leg length and BMI), these models may apply to other populations. However, considering the strong correlation between step length and the consistency of our results with previous reports, we assume that the selected variables in the models may remain significant factors for estimating step length. Lastly, other methods such as DXA may better reflect more accurate skeletal muscle and fat mass, although BIA and sEMG have their own merits in terms of cost-effectiveness (both BIA and sEMG) and availability of measurement during actual function (sEMG).

## 5. Conclusions

This study revealed that the RMS value at maximal knee flexion strength, height, age, and BFM are predictors of individual step length. Strengthening knee flexor function and reducing BFM may help improve step length.

## Figures and Tables

**Table 1:** Participant demographics and the results of body impedance analysis and surface electromyography measurements.

Variables (*n* = 179)	Mean ± SD	Min	Max	Median
Age (years)	60.0 ± 11.1	41	79	60.5
Sex (M:F)	82:97	-	-	-
Height (cm)	162.7 ± 8.6	140	182	162.25
BMI (kg/m^2^)	24.6 ± 3.0	18.0	32.9	24.0
Average step length (cm)	62.8 ± 6.8	41.0	83.1	65.6
ASM (kg)/(height, m)^2^	7.1 ± 1.1	5.2	9.4	7.0
BFM (kg)	18.9 ± 5.9	5.8	38.3	18.2
Average RMS (µV)				
Knee flexion	321.7 ± 128.8	84.4	789.2	310.8
Knee extension	233.0 ± 100.4	41.0	558.2	218.9

Data (except for sex) are presented as mean ± SD (standard deviation); Min, minimum; Max, maximum; BMI, body mass index; ASM, appendicular skeletal muscle mass; BFM, body fat mass; RMS, root mean square.

**Table 2:** Participant demographics and results of body impedance analysis and surface electromyography measurements by age group and sex.

Age Group	40s		50s		60s		70s		*p*-Value between Age Groups
*n*	40		45		49		45		
	M (*n* = 16)	W (*n* = 24)	M (*n* = 20)	W (*n* = 25)	M (*n* = 24)	W (*n* = 25)	M (*n* = 22)	W (*n* = 23)	
Age (years)	45.1 ± 2.6		54.1 ± 2.9		64.4 ± 3.0		74.1 ± 2.6		-
	44.1 ± 2.7	45.7 ± 2.4	54.6 ± 3.0	53.7 ± 2.9	65.1 ± 2.6	63.6 ± 3.2	75.2 ± 2.7	73.0 ± 2.1	
*p*-Value between sexes	NS		NS		NS		0.0043		
Height (cm)	165.9 ± 7.8		164.1 ± 8.1		160.6 ± 9.1		161.0 ± 8.4		0.0093
	173.1 ± 4.0	161.0 ± 5.5	171.4 ± 5.2	158.4 ± 4.5	167.5 ± 6.3	154.0 ± 5.7	166.6 ± 5.3	155.7 ± 7.5	
*p*-Value between sexes	<0.0001		<0.0001		<0.0001		<0.0001		
BMI (kg/m^2^)	24.2 ± 3.4		24.8 ± 3.4		25.2 ± 2.5		23.9 ± 2.6		NS
	26.0 ± 3.0	23.0 ± 3.2	26.3 ± 2.3	23.6 ± 3.7	25.5 ± 2.2	24.9 ± 2.7	23.4 ± 2.8	24.5 ± 2.3	
*p*-Value between sexes	0.0053		0.0083		NS		NS		
Average step length (cm)	64.4 ± 4.8		64.8 ± 5.6		62.0 ± 7.0		60.2 ± 8.3		0.0034
	65.9 ± 4.2	63.4 ± 5.0	66.8 ± 6.0	63.3 ± 4.8	64.3 ± 7.3	59.7 ± 6.1	62.8 ± 8.4	57.8 ± 7.5	
*p*-Value between sexes	NS		0.0343		0.0183		0.0412		
ASM (kg/m^2^)	7.3 ± 1.1		7.3 ± 1.2		7.2 ± 1.1		6.9 ± 0.9		NS
	8.4 ± 0.5	6.5 ± 0.6	8.4 ± 0.6	6.3 ± 0.6	8.0 ± 0.8	6.4 ± 0.7	7.5 ± 0.7	6.3 ± 0.5	
*p*-Value between sexes	<0.0001		<0.0001		<0.0001		<0.0001		
BFM (kg)	18.9 ± 6.4		19.3 ± 5.8		19.0 ± 5.4		18.4 ± 6.2		NS
	19.2 ± 6.6	18.7 ± 6.4	18.9 ± 4.1	19.7 ± 6.8	18.0 ± 5.2	19.9 ± 5.4	15.6 ± 5.9	21.1 ± 5.3	
*p*-Value between sexes	NS		NS		NS		0.0017		
Average RMS: knee flexion (µV)	348.4 ± 165.1		334.2 ± 123.9		345.1 ± 112.4		260.2 ± 91.8		0.0024
	409.6 ± 165.5	307.6 ± 155.0	356.0 ± 117.1	316.7 ± 128.8	386.6 ± 115.4	305.2 ± 95.6	310.7 ± 97.1	211.8 ± 53.3	
*p*-Value between sexes	NS		NS		0.0098		0.0001		
Average RMS: knee extension (µV)	246.0 ± 99.8		265.2 ±105.4		227.9 ± 87.4		194.8 ± 98.9		0.0068
	267.2 ± 112.0	231.9 ± 90.4	282.9 ± 103.8	251.1 ± 106.5	252.5 ± 102.9	204.3 ± 62.7	214.2 ± 120.1	176.3 ± 71.2	
*p*-Value between sexes	NS		NS		NS		NS		

Data are presented as mean ± SD; 40s, age 40–49 years; 50s, age 50–59 years; 60s, age 60–69 years; 70s, age 70–79 years; M, men; W, women; NS, not significant; BMI, body mass index; ASM, appendicular skeletal muscle mass; BFM, body fat mass; RMS, root mean square.

**Table 3:** Differences in the step length, height, and average RMS value at knee flexion between men and women.

	Men (*n* = 82)	Women (*n* = 97)	*p*-Value
Average step length (cm)	64.83 ± 1.52	61.09 ± 1.27	<0.001
Height (cm)	169.30 ± 1.29	157.26 ± 1.28	<0.001
average RMS: knee flexion (µV)	363.24 ± 27.67	286.62 ± 24.36	<0.001
BFM (kg)	17.79 ± 1.22	19.85 ± 1.21	NS

Data are presented as mean ± SD; RMS, root mean square; BFM, body fat mass; NS, not significant.

**Table 4:** The three-step length estimation models using age, height, BFM, and RMS value at maximal strength knee flexion.

Model	Variables	R^2^	Adjusted R^2^	C*_p_*	AIC	BIC	Regression Coefficient	*p*-Value
	RMS at maximal strength knee flexion	0.244	0.235	7.28	642.18	644.14	0.018	<0.001
1	height						0.23	<0.001
	(constant)						19.506	0.023
	Age	0.198	0.185	3.26	654.80	657.02	−0.115	0.008
2	Height						0.238	<0.001
	BFM						−0.235	0.003
	(constant)						35.435	<0.001
3	Age	0.158	0.148	4.43	661.61	663.66	−0.109	0.013
	Height						0.251	<0.001
	(constant)						28.444	0.005

RMS, root mean square; BFM, body fat mass; C*_p_*, Mallows’ Cp; AIC, Akaike information criterion; BIC, Bayesian information criterion.

## Data Availability

The data presented in this study are available upon reasonable request from the corresponding author. The data are not publicly available because of privacy concerns.

## Supplementary Materials

The following supporting information can be downloaded at: https://www.mdpi.com/article/10.3390/s22155686/s1. Figure S1. The representative figures for sEMG during the maximal effort knee flexion (A) and extension (B). Figure S2: Scatter plots and results of the simple linear regression analysis between the average step length and variables: (A–G). Figure S3: Linear regression between the average step length and gait velocity (R^2^ = 0.6860, *p* < 0.0001). Figure S4: Linear regression between the maximal flexor strength and RMS at maximal knee flexion (R^2^ = 0.1549, *p* < 0.0001).
